# Advances in the Uptake and Transport Mechanisms and QTLs Mapping of Cadmium in Rice

**DOI:** 10.3390/ijms20143417

**Published:** 2019-07-11

**Authors:** Jingguang Chen, Wenli Zou, Lijun Meng, Xiaorong Fan, Guohua Xu, Guoyou Ye

**Affiliations:** 1 CAAS-IRRI Joint Laboratory for Genomics-Assisted Germplasm Enhancement, Agricultural Genomics Institute in Shenzhen, Chinese Academy of Agricultural Sciences, Shenzhen 518120, China; 2 College of Resources and Environmental Sciences, Nanjing Agricultural University, Nanjing 210095, China; 3 Strategic Innovation Platform, International Rice Research Institute, DAPO Box 7777, Metro Manila 1226, Philippines

**Keywords:** cadmium accumulation, absorption and transport, QTL location, mapping population, rice (*Oryza sativa* L.)

## Abstract

Cadmium (Cd), as a heavy metal, presents substantial biological toxicity and has harmful effects on human health. To lower the ingress levels of human Cd, it is necessary for Cd content in food crops to be reduced, which is of considerable significance for ensuring food safety. This review will summarize the genetic traits of Cd accumulation in rice and examine the mechanism of Cd uptake and translocation in rice. The status of genes related to Cd stress and Cd accumulation in rice in recent years will be summarized, and the genes related to Cd accumulation in rice will be classified according to their functions. In addition, an overview of quantitative trait loci (QTLs) mapping populations in rice will be introduced, aiming to provide a theoretical reference for the breeding of rice varieties with low Cd accumulation. Finally, existing problems and prospects will be put forward.

## 1. Introduction

Cadmium (Cd) is a soil contaminant and with a high mobility in living organisms, and is characterized as a toxic heavy metal [1,2]. In China, about 2.786 × 10^9^ m^2^ of agricultural land is contaminated by Cd [3]. Frequent applications of nitrogen fertilizer in the agricultural land of many areas of China have resulted in more acidic soil, and acidic soil means that cadmium is more easily absorbed by plants [4]. Rice (*Oryza sativa* L.) is the main food for more than half of the world’s population. Cd is easily transferred from soil to rice and accumulates in rice plants and grains [2,3], and is then enriched in the human body through the food chain, thereby threatening human health [5,6,7], and causing effects such as anemia, cancer, heart failure, hypertension, cerebral infarction, proteinuria, severe lung damage, eye cataract formation, osteoporosis, emphysema, and renal insufficiency [8,9]. It is worth mentioning that Itai-itai disease, which occurred in Japan in the 1950s, was caused by the long-term intake of cadmium-contaminated rice [10]. On average, weekly Cd accumulation was as high as 3–4 mg kg^−1^ body weight in Japan at that time [11]. Between 1990 and 2015, the average dietary Cd intake of the general population more than doubled in China [12,13]. Therefore, reducing Cd uptake by crops, especially rice, is of great significance to food safety and human health.

The purpose of this review is to explore the mechanism of cadmium uptake and transport and the genetic characteristics of Cd accumulation in rice, and to summarize the research status of genes and QTLs related to cadmium stress and cadmium accumulation in rice. It has important guiding significance for breeding high-quality rice varieties with a low accumulation of Cd in grain and the safe production of rice in mild and moderate Cd-contaminated soil.

## 2. Toxic Effects of Cadmium Exposure on Rice

Cd stress seriously affects rice germination and growth [2,3,14,15,16,17,18], and it was found that excessive Cd exposure can not only significantly decrease the rice seed germination rate [14], but also cause chlorosis and necrosis in rice plants during the vegetative stage [19,20]. Cd stress causes severe physical and physiological changes in rice plants as it causes a reduction in the length; width; and number of roots, shoots, and leaves. Furthermore, chlorophyll contents, stomatal conductance, and the water use efficiency of rice are also significantly affected [3,17,18,21,22,23]. Cd also affects the absorption and transport of essential nutrients in rice [15,16,18,19,20]. Additionally, Cd can be transported to rice grains, reducing their yield, quality, and nutrients [15,16,24,25,26,27]. In general, Cd stress inhibits rice growth [18,28,29,30].

Rice possesses some tolerance mechanisms to cadmium at physiological and molecular levels [31,32,33,34,35]. As root cell walls of the outermost layer have direct contact with the soil solution, this protects the protoplasts against Cd toxicity [36,37,38]. Furthermore, plants reduce Cd translocation to the shoots by immobilizing Cd in the cell walls and vacuoles of root cells, thus reducing their sensitivity and the harm of Cd to another cellular organelle [39,40,41,42]. Several adenosine triphosphate (ATP)-binding cassette (ABC) proteins have been reported to mediate vacuolar compartmentation of Cd-glutathione and/or phytochelatin (PC) conjugates in *Arabidopsis thaliana* [43,44]. Rice *OsPDR5/ABCG43* is likely to encode ABC-type protein functions in Cd extrusion from the cytoplasm [45]. Overexpression of Cd transporter OsHMA3 located in vacuole membranes in rice roots can increase the tolerance of rice to Cd and reduce the accumulation of Cd in grains [46,47]. Exudates of roots contain metal chelators which play a role in the adjustment of the rhizosphere pH and the metal chelating process [48]. Most of the chelated toxic metals inside plants target vacuoles through metal detoxification processes [38,49]. Organic acids secreted from roots, e.g., malate, citrate, etc., are involved in metal uptake, the long-distance transport of metal, and the transport of metal into vacuoles [50,51]. It was found that chelators play a crucial role in keeping Cd in the rice roots and form a barrier in Cd translocation [52].

Cd stress can induce plants to enhance their antioxidant defense system and regulate ion homeostasis to improve their tolerance to Cd [32,53,54,55,56,57,58]. For example, Cd stress can induce plants to increase the production of glutathione (GSH), abscisic acid (ABA), salicylic acid (SA), jasmonic acid (JA), and nitric oxide (NO) [59,60,61,62,63,64,65]. Mitogen-activated protein kinase OsWJUMK1, OsMSRMK2, OsMSRMK3, and OsMAPK2 can affect rice root growth under Cd stress by regulating auxin signal changes [66,67,68,69]. Auxin transporter *OsAUX1* has been reported to be involved in root development and the Cd stress response in rice [34]. In addition, the concentration of iron and cadmium was positively correlated during rice seedling growth [70]. It has been reported that increasing the supply of boron, iron, zinc, silicon, or magnesium can reduce the accumulation and toxicity of cadmium in rice [71,72,73,74,75,76].

In addition, some genes related to Cd stress have been reported in rice (Table 1). *OsHMA9* is a copper efflux protein located in the plasma membrane, which may have a cadmium efflux function to excrete Cd from root cells and reduce Cd accumulation in rice [77]. Knock-out of the low cadmium gene (*LCD*) reduced the accumulation of cadmium and increased the growth of rice under the condition of an excessive cadmium supply, and LCD may be a protein related to cadmium homeostasis [78]. Overexpression of *OsCDT1* can increase the growth of *Arabidopsis thaliana* under cadmium treatment; the cysteine-rich peptide encoded by *OsCDT1* is possibly involved in rice Cd tolerance [79]. *OsCLT1* probably mediates the export of γ-glutamylcysteine and glutathione from plastids to the cytoplasm, which in turn affects As and Cd detoxification in rice [80].

## 3. Uptake and Transport Pathway of Cd in Rice

**Figure 1:**
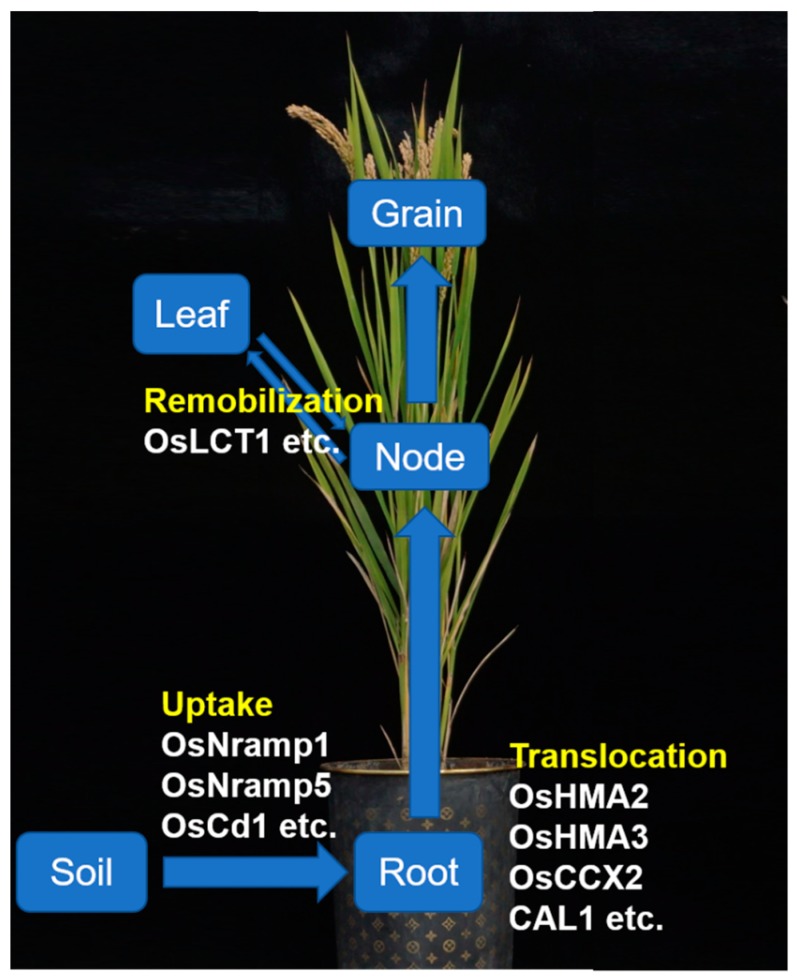
A schematic of cadmium transport from the soil to grains in rice. Cadmium is absorbed from the soil by the roots, and *OsNramp1*, *OsNramp5*, and *OsCd1* mediate this process. *OsHMA3* plays a key role in cadmium segregation to vacuoles in root cells and thus negatively regulates cadmium xylem loading. *OsHMA2*, *OsCCX2*, and *CAL1* regulate cadmium transport to the xylem. *OsLCT1* contributes to cadmium remobilization from leaf blades via the phloem and is likely to play a part in intervascular cadmium transfer at nodes.

Cadmium is transported from the roots to shoots and then to grains through four steps: (i) uptake by roots; (ii) transportation to shoots through loading to the xylem; (iii) distribution and transportation through nodes; and (iv) transportation to grains through the phloem from leaf blades (Figure 1).

### 3.1. Functional Analysis of Related Genes

Cd can enter rice plants through the uptake mechanism of essential elements such as Mn, Zn, and Fe, etc. [106,107,119]. Fe^2+^ transporters *OsIRT1* and *OsIRT2* display Cd^2+^ influx activity in yeast, which indicates that *OsIRT1* and *OsIRT2* may play a role in cadmium uptake in the root system [95,120]. Overexpression of *OsIRT1* significantly increased the accumulation of Cd in roots and shoots in Murashige & Skoog (MS) medium containing excess Cd, but no obvious phenotype was observed under field conditions, suggesting that *OsIRT1* may be involved in cadmium uptake in roots, but its contribution is largely affected by environmental conditions [96]. Oryza sativa Natural Resistance-Associated Macrophage Protein 5 (OsNramp5), located at the plasma membrane of root cells, was found to be the major transporter of Cd uptake in rice roots, responsible for the transport of Cd from the soil solution to the root cells [106,107]. OsNramp5 is also an Mn transporter, and the knock-out of *OsNramp5* can significantly reduce the uptake and accumulation of cadmium in grains, but also lead to the decrease of growth and yield due to manganese deficiency [107,108,109]. Recently, Liu et al. [121] located a major QTL, *qGMN7.1*, according to the Mn concentration in the grains of a recombinant inbred line (RILS) crossed between 93–11 (low grain Mn) and PA64s (high grain Mn). Fine mapping delimited *qGMN7.1* to a 49.3 kb region containing *OsNRAMP5*, and sequence variations in the *OsNRAMP5* promoter caused changes in its transcript level and in grain Mn levels. Tang et al. [110] reported that a series of new indica rice lines with low cadmium accumulation were developed by knocking out the metal transporter *OsNramp5* using the CRISPR/CAS9 system. OsNRAMP1, located on the plasma membrane, also exhibits the activity of Cd transport, and participates in the uptake and transport of Cd in root cells [111,112]. OsZIP1, a zinc-regulated/iron-regulated transporter-like protein, expression in yeast can enhance its sensitivity to Cd [84], and the overexpression of *OsZIP6* can increase the Cd uptake in *X. laevis oocytes* [98].

After root absorption, xylem-mediated Cd translocation from the roots to shoots is the main factor determining the cadmium accumulation in shoots [122]. *OsHMA2* and *OsHMA3* were reported to play a role in this process [46,103,123,124]. *OsHMA2* participates in the transport of Cd from the roots to shoots and plays an important role in controlling the distribution of Cd through the phloem to developing tissues [103,104,123]. Compared with wild-type (WT) samples, the Cd concentration in the shoots of an *oshma2* mutant was significantly lower [104]. OsHMA3 plays a role in the vacuolar sequestration of Cd in root cells, the overexpression of *OsHMA3* reduces the Cd load in the xylem and Cd accumulation in shoots, and the functional deficiency of *OsHMA3* results in very high root-to-shoot Cd translocation in rice [46,47,105,125]. Recent reports showed that OsCCX2, a putative cation/calcium (Ca) exchanger, was localized in the plasma membrane and plays an important role in Cd transport by impacting Cd root-to-shoot translocation and the Cd distribution in the shoot tissues, and the knock-out of *OsCCX2* resulted in a significant Cd reduction in the grains [94]. Tan et al. [99] reported that *OsZIP7* plays a key role in xylem-loading in roots and inter-vascular transfer in nodes to deliver Zn and Cd upward in rice.

**Figure 2:**
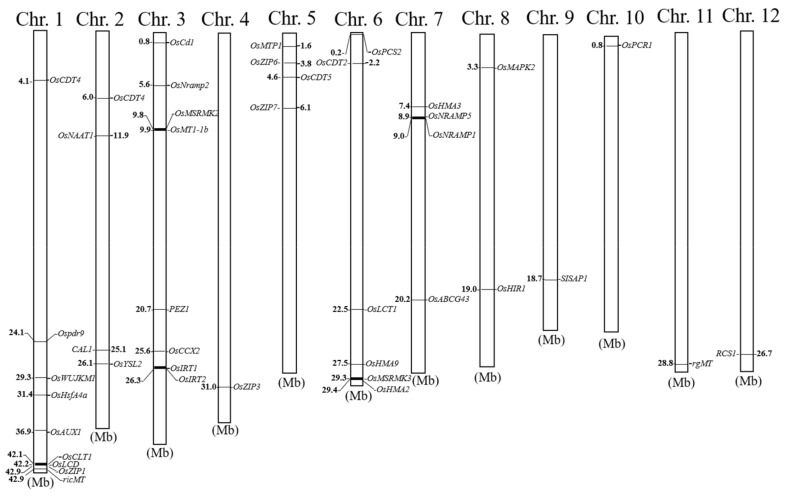
Positions of cloned cadmium stress-related genes in rice chromosomes.

Nodes are the central organ of Cd transport from the xylem to phloem, and play an important role in Cd transport to grains [126,127,128]. OsLCT1 is a Cd-efflux transporter on the plasma-membrane involved in phloem Cd transport [101]. *OsLCT1* expression was higher in leaf blades and nodes during the reproductive stage, especially in node I. Compared with wild-type (WT), the Cd concentration in phloem exudates and in grains of *OsLCT1* RNAi plants decreased significantly, although the Cd concentration in xylem sap did not differ. These results suggest that *OsLCT1* in leaf blades functions in Cd remobilization by the phloem, and in node I, *OsLCT1* is likely to play a part in intervascular Cd transfer from enlarged large vascular bundles to diffused vascular bundles, which connect to the panicle [101,102]). The positions of cloned cadmium stress-related genes in rice chromosomes are shown in Figure 2.

### 3.2. Location of Related QTLs

Rice varieties show obvious genetic variation in terms of their cadmium accumulation ability, which is a valuable resource for dissecting functional alleles and genetic improvement [19,20,25]. However, only a few quantitative trait loci (QTLs) related to cadmium accumulation in rice have been reported. *OsHMA3*, *CAL1* (Cd Accumulation in Leaf 1), and *OsCd1* are the only Cd-related QTLs cloned so far. OsHMA3 encodes a cadmium transporter located in the vacuole membrane, which transports cadmium into vacuoles for sequestration [105]. Loss of OsHMA3 function significantly increased cadmium transport to rice shoots and grains [101,129]. On the other hand, the overexpression of *OsHMA3* can increase the tolerance of rice to Cd and reduce Cd accumulation in grains [46,105,119]. *CAL1* (cadmium accumulation in leaf 1) was identified and cloned by Luo et al. [88] as a quantitative trait locus (QTL) in rice, which explained 13% of the variation in leaf cadmium concentration in a doubled haploid population. *CAL1* regulates the root-to-shoot translocation of cadmium via the xylem vessels, and knockout mutants of *CAL1* significantly reduced the concentration of cadmium in rice leaves [88]. Yan et al. [90] discovered that the gene *OsCd1* belongs to the major facilitator superfamily through genome-wide association studies (GWAS), which was associated with divergence in rice grain Cd accumulation. Interestingly, the natural variation *OsCd1^V449^* in *Japonica*, which is associated with a reduced Cd transport ability and decreased grain Cd accumulation, shows a potential value in low-Cd rice selection [90].

A series of QTLs related to rice varieties that control the Cd concentration in rice have been reported (Table 2). Ishikawa et al. [130] obtained a mapping population consisting of 85 back-cross inbred lines (BIL) from hybridization between a low-cadmium-accumulation variety of *Japonica* rice (Sasanishiki) and a high-cadmium-accumulation variety of *Indica* rice (Habataki). Two QTLs were located on chromosomes 2 and 7, separately, with an increased cadmium concentration in grains. *qGCd7* plays an important role in increasing the cadmium concentration in grains, which can explain 35.5% of phenotypic variation [130]. Kashiwagi et al. [131] identified two QTLs, known as *qcd4–1* and *qcd4–2*, affecting the cadmium concentration in shoots. Sato et al. [132] reported two QTLs controlling the cadmium concentration in brown rice: *qLCdG11* explained 9.4%–12.9% of phenotypic variation and *qLCdG3* explained 8.3%–13.9% of phenotypic variation. Yan et al. [133] constructed an recombinant inbred lines (RIL) population of F7 to identify Cd accumulation and distribution. A total of five main effect QTLs (*scc10* was correlated with Cd accumulation in shoots; *gcc3, gcc9,* and *gcc11* with Cd accumulation in grains; and *sgr5* with the Cd distribution ratio in shoots and roots) were detected. Among them, *sgr5* had the greatest effect on the distribution of Cd in grains. Abe et al. [134] used a population consisting of 46 chromosome segment substitution lines (CSSL) to identify eight QTLs related to the grain cadmium content by single-label analysis using ANOVA. The result showed that *qlGCd3* had a high F-test value. A recombinant inbred population derived from Xiang 743/Katy was grown in Cd-polluted fields and used to map the QTLs for Cd accumulation in rice grains, and two QTLs, *qCd-2* and *qCd-7*, were identified in 2014 and 2015 [135]. Liu et al. [136] used 276 accessions with 416 K single nucleotide polymorphisms (SNPs) and performed a genome-wide association analysis of grain Cd concentrations in rice grown in heavily multi-contaminated farmlands, and 17 QTLs were found to be responsible for the grain Cd concentration.

## 4. Future Perspectives

Cadmium is a kind of heavy metal that presents extreme biological toxicity. Cd accumulated in rice can enter the food chain, thereby threatening human health [5,6,7]. Cadmium in rice can be reduced by agronomic practices (including soil amendments, fertilizer management, water management, and tillage management) and bioremediation (including phytoremediation and microbial remediation) [18,145,146,147,148,149,150]. In addition, understanding the mechanism of cadmium translocation and the factors affecting cadmium accumulation in rice are also important for formulating effective strategies to reduce cadmium accumulation in rice. In recent years, some genes related to cadmium transport in rice have been studied, and significant progress has been made in understanding the mechanism of cadmium uptake and transport. In order to understand the mechanism of cadmium transport in rice, it is necessary to identify more unknown transporters or other molecules.

Biotechnology offers a promising approach to reducing the Cd content in rice grains. Mutations of the *OsNramp5* gene result in obvious decreases in Cd uptake in roots and Cd accumulation in rice grains [106,108,151]. Using the CRISPR/Cas9 gene editing technology to knock out *OsNramp5* in both parental lines, Tang et al. [110] generated a hybrid rice cultivar that accumulated very low levels of Cd in the grain. Another target for gene editing is *OsLCT1*, which is involved in the phloem transport of Cd from the vegetative tissues to the grains [101]. Knockdown of this gene by RNAi reduced the grain Cd concentration by 30%–50% [101]. Overexpression of functional *OsHMA3* in *Nipponbare* decreased Cd translocation and Cd accumulation in rice grains [46,105]. Overexpression of *OsHMA3* is a highly effective method for reducing Cd accumulation in *Indica* rice, and rice grains produced using this approach are almost Cd-free, with little effect on the grain yield or essential micronutrient concentrations [152].

However, commercial transgenic rice is not commonly accepted by the general public and prohibited in many countries. Ishikawa et al. [151] produced three rice mutants by carbon ion-beam irradiation, where cadmium was hardly detected in mutant seeds when planted in cadmium-contaminated paddy fields and there was no significant difference between the mutant and wild-type (WT) in agronomic traits, which could be directly applied to breeding projects. Another possible strategy is marker-assisted breeding, which uses molecular markers to track the genetic composition of rice and bred rice varieties. For example, identifying a low-cadmium-related QTL and then introducing it into high-cadmium cultivars might be a viable approach [122]. However, only a few of QTLs related to cadmium accumulation in rice have been cloned [90,105], and the natural allele variation of grain cadmium accumulation differences among rice varieties has not been fully explored. Further research is necessary to clone more QTLs for controlling grain Cd accumulation, thus providing tools for the marker-assisted molecular breeding of rice cultivars with a low accumulation of Cd in grains.

## Figures and Tables

**Table 1:** Genes of Rice Reported to be Regulated During Cadmium (Cd)-Exposure.

Gene	Chr.	Physical Location (bp)	Gene Name	Function	Reference
*OsCDT3*	1	4066623–4067218	Encoding a Cys-rich peptide	Cd uptake inhibitor	[79]
*Ospdr9*	1	24075065–24082181	Multidrug resistance ABC transporter	Redox protection in Cd stress	[81]
*OsWJUMK1*	1	29398191–29402466	Mitogen-activated protein kinase	Cd signal	[82]
*OsHsfA4a*	1	31370413–31372729	Heat shock transcription factor gene	Cd tolerance	[83]
*OsAUX1*	1	36998334–37004685	Auxin transport protein	Root development and Cd stress response	[34]
*OsCLT1*	1	42086484–42095424	CRT-like transporter 1	Cd tolerance	[80]
*OsLCD*	1	42162592–42166462	Low cadmium	Cd tolerance and accumulation	[78]
*OsZIP1*	1	42905566–42907474	Zinc- and iron-regulated transporter	Cd and Zn transport	[84,85]
*ricMT*	1	43047164–43047861	Metallothionein gene	Cd tolerance	[86]
*OsCDT4*	2	6078179–6079111	Encoding a Cys-rich peptide	Cd uptake inhibitor	[79]
*OsNAAT1*	2	11997094–12002633	Nicotinamide aminotransferase gene	Cd accumulation	[87]
*CAL1*	2	25190487–25191188	defensin-like protein	Cd accumulation in leaf	[88]
*OsYSL2*	2	26170387–26174970	Metal-nicotinamide transporter	Cd translocation	[89]
*OsCd1*	3	842577–846408	Major facilitator superfamily	Cd uptake	[90]
*OsNramp2*	3	5655157–5659147	Natural resistance-associated macrophage protein	Cd transporter, Cd accumulation	[91]
*OsMSRMK2*	3	9847700–9850473	Mitogen-activated protein kinase	Cd signal	[67]
*OsMTI-1b*	3	9957335–9958362	Metallothionein-like protein 1B	Cd tolerance	[92]
*PEZ1*	3	20793053–20799805	Phenol efflux protein	Cd accumulation	[93]
*OsCDT1/OsCCX2*	3	25613825–25616179	Cation/calcium (Ca) exchanger 2	Cd tolerance and translocation	[79,94]
*OsIRT2*	3	26276301–26277206	Iron-regulated transporter	Cd and Fe transporter	[95]
*OsIRT1*	3	26286156–26292023	Iron-regulated transporter	Cd and Fe transporter	[95,96]
*OsZIP3*	4	31078200–31080734	Zinc- and iron-regulated transporter	Cd accumulation	[84]
*OsMTP1*	5	1675488–1679056	Metal tolerance protein gene	Cd translocation	[97]
*OsZIP6*	5	3807974–3810752	Zinc- and iron-regulated transporter	Cd transport	[98]
*OsCDT5*	5	4665325–4667853	Encoding a Cys-rich peptide	Cd uptake inhibitor	[79]
*OsZIP7*	5	6090801–6094068	Zinc- and iron-regulated transporter	Cd and Zn accumulation	[99]
*OsPCS2*	6	167367–174319	Plant chelatase synthase 2	Cd tolerance	[100]
*OsCDT2*	6	2261681–2263972	Encoding a Cys-rich peptide	Cd uptake inhibitor	[79]
*OsLCT1*	6	22566775–22571982	Low affinity cation transporter	Cd transporter in phloem	[101,102]
*OsHMA9*	6	27517100–27523604	P-Type Heavy Metal ATPase	Cd efflux	[77]
*OsMSRMK3*	6	29398191–29402466	Mitogen-activated protein kinase	Cd signal	[82]
*OsHMA2*	6	29477949–29480905	P-Type Heavy Metal ATPase	Cd and Zn translocation	[103,104]
*OsHMA3*	7	7405745–7409553	P-Type Heavy Metal ATPase	Sequestration of Cd in root	[46,47,105]
*OsNramp5*	7	8871436–8878905	Natural resistance-associated macrophage protein	Cd, Mn, and Fe transporters	[106,107,108,109,110]
*OsNramp1*	7	8966025–8970882	Natural resistance-associated macrophage protein	Cd and Fe transporters	[111,112,113]
*OsABCG43*	7	20214025–20218702	ATP-binding cassette transporter	Cd compartmentalization	[45]
*OsMAPK2*	8	3307520–3310590	Mitogen-activated protein kinase	Cd signal	[68]
*OsHIR1*	8	19011814–19015998	Heavy metal-induced RING E3 ligase 1	Cd uptake	[114]
*SISAP1*	9	18760704–18761836	Subspecies indica stress-associated protein gene	Cd tolerance	[115]
*OsPCR1*	10	826309–824623	Plant cadmium resistance 1	Cd tolerance	[116]
*rgMT*	11	28827746–28828439	Metallothionein-like protein	Cd tolerance	[117]
*RCS1*	12	26698650–26703087	Cytosolic cysteine synthase gene	Cd complexation via sulfur	[118]

**Table 2:** Quantitative Trait Loci (QTLs) of Rice Reported to be Regulated during Cadmium (Cd)-Exposure.

Stage	Parent Sources	Population	Marker	Trait	Chr.	QTL	Reference
Seedling stage	Tainan1/Chunjiang06	119 DH, 3651 BC3F3	RFLP	Cd accumulation in leaves	2	*CAL1*	[88]
Seedling stage	Nipponbare/Anjana Dhan	965 F2	SSR	Cd concentration in shoots	7	*OsHMA3*	[105]
Seedling stage	SNU-SG1/Suwon490	91 RIL	124 SSR	Cd concentration in shoots	10	*scc10*	[133]
Seedling stage	Koshihikari/LAC23	46 CSSLs	345 SNP	Cd concentration in shoots	3	*glGCd3*	[134]
Seedling stage	Anjana Dhan/Nipponbare	177 F2	SSR	Root-to-shoot Cd translocation	7	*qCdT7*	[137]
Seedling stage	Badari Dhan/Shwe War	184 F2	141 SSR	Cd concentration in shoots	2,5,11	—	[138]
Seedling stage	JX17/ZYQ8	127 DH	160 RFLP,83 SSR	Shoot/root rate of Cd concentration	3	*qSRR3*	[139]
Seedling stage	JX17/ZYQ8	127 DH	160 RFLP,83 SSR	Cd concentration in roots and shoots	6,7	*qCDS7, qCDR6.1, qCDR6.2*	[139]
Seedling stage	Azucena/Bala	79 RIL	164 SSR	Cd concentration in leaves	1,3,6	*qCd1, qCd3, qCd6,*	[140]
Bfore heading	Kasalath/Nipponbare	98 BILs	RFLP and SSR	Cd concentration in leaves and culms	4,11	*qcd4–1, qcd4–2, qcd11*	[131]
Mature period	Sasanishiki/Habataki	85 BIL	SSR	Cd accumulation in grains	2,7	*qGCd7*	[130]
Mature period	Fukuhibiki/LAC23	126 RIL	454 SNP	Cd accumulation in grains	3,11	*gLCdG3, gLCdG11*	[132]
Mature period	SNU-SG1/Suwon490	91 RIL	124 SSR	Cd accumulation in grains	3,5,9,11	*gcc3, sgr5, gcc9, gcc11*	[133]
Mature period	Xiang 743/Katy	115 RIL,	SSR	Cd accumulation in grains	2,7	*qCd-2, qCd-7*	[135]
Mature period	Kasalath/Koshihikari	39CSSL	129 RFLP	Cd accumulation in grains	3,6,8	—	[141]
Mature period	Koshihikari/Jarjan	103 BIL	169 SSR	Cd accumulation in grains	7	—	[142]
Mature period	JX17/ZYQ8	127 DH	160 RFLP,83 SSR	Cd accumulation in grains	3,6	*gCdc3, gCdc6*	[143]
Mature period		127 rice cultivars	GWAS	Cd accumulation in grains	3	*OsCd1*	[90]
Mature period		378 rice cultivars	GWAS	Cd accumulation in grains	3, 5	*qCd3, qCd5.1, qCd5.2*	[144]

## References

[1] He S.Y., He Z.L., Yang X.E., Stoffella P.J., Baligar V.C. (2015). Soil biogeochemistry, plant physiology, and phytoremediation of cadmium-contaminated soils. Adv. Agron..

[2] Song W.E., Chen S.B., Liu J.F., Chen L., Song N.N., Li N., Liu B. (2015). Variation of Cd concentration in various rice cultivars and derivation of cadmium toxicity thresholds for paddy soil by species-sensitivity distribution. J. Integr. Agric..

[3] Liu F., Liu X.N., Ding C., Wu L. (2015). The dynamic simulation of rice growth parameters under cadmium stress with the assimilation of multi-period spectral indices and crop model. Field Crop. Res..

[4] Zhao K.L., Fu W.J., Ye Z.Q., Zhang C.S. (2015). Contamination and spatial variation of heavy metals in the soil-rice system in Nanxun County, Southeastern China. Int. J. Environ. Res. Public Health..

[5] Xie P.P., Deng J.W., Zhang H.M., Ma Y.H., Cao D.J., Ma R.X., Liu R.J., Liu C., Liang Y.G. (2015). Effects of cadmium on bioaccumulation and biochemical stress response in rice (*Oryza sativa* L.). Ecotox. Environ. Safe..

[6] Xue D.W., Jiang H., Deng X.X., Zhang X.Q., Wang H., Xu X.B., Hu J., Zeng D.L., Guo L.B., Qian Q. (2014). Comparative proteomic analysis provides new insights into cadmium accumulation in rice grain under cadmium stress. J. Hazard. Mater..

[7] Aziz R., Rafiq M.T., Li T., Liu D., He Z., Stoffella P.J., Sun K., Xiaoe Y. (2015). Uptake of cadmium by rice grown on contaminated soils and its bioavailability/toxicity in human cell lines (Caco-2/HL-7702). J. Agric. Food Chem..

[8] Godt J., Scheidig F., Grosse-Siestrup C., Esche V., Brandenburg P., Reich A., Groneberg D.A. (2006). The toxicity of cadmium and resulting hazards for human health. J. Occup Med. Toxicol..

[9] Satarug S., Baker J.R., Urbenjapol S., Haswell-Elkins M., Reilly P.E., Williams D.J., Moore M.R. (2003). A global perspective on cadmium pollution and toxicity in non-occupationally exposed population. Toxicol Lett..

[10] Horiguchi H., Teranishi H., Niiya K., Aoshima K., Katoh T., Sakuragawa N., Kasuya M. (1994). Hypoproduction of erythropoietin contributes to anemia in chronic cadmium intoxication — clinical-study on itai-itai disease in Japan. Arch. Toxicol..

[11] Tsukahara T., Ezaki T., Moriguchi J., Furuki K., Shimbo S., Matsuda-Inoguchi N., Ikeda M. (2003). Rice as the most influential source of cadmium intake among general Japanese population. Sci. Total Environ..

[12] Song Y., Wang Y., Mao W., Sui H., Yong L., Yang D., Jiang D., Zhang L., Gong Y. (2017). Dietary cadmium exposure assessment among the Chinese population. PLoS ONE.

[13] Chen H., Yang X., Wang P., Wang Z., Li M., Zhao F.J. (2018). Dietary cadmium intake from rice and vegetables and potential health risk: A case study in Xiangtan, southern China. Sci. Total Environ..

[14] Ahsan N., Lee S.H., Lee D.G., Lee H., Lee S.W., Bahk J.D., Lee B.H. (2007). Physiological and protein profiles alternation of germinating rice seedlings exposed to acute cadmiumtoxicity. C. R. Biol..

[15] Li B., Wang X., Qi X., Huang L., Ye Z. (2012). Identification of rice cultivars with low brown rice mixed cadmium and lead contents and their interactions with the micronutrients iron, zinc, nickel and manganese. J. Environ. Sci..

[16] Li S., Yu J., Zhu M., Zhao F., Luan S. (2012). Cadmium impairs ion homeostasis by altering K^+^ and Ca^2+^ channel activities in rice root hair cells. Plant Cell Environ..

[17] Wang Y., Jiang X., Li K., Wu M., Zhang R., Zhang L., Chen G. (2014). Photosynthetic responses of *Oryza sativa* L. seedlings to cadmium stress: Physiological, biochemical and ultrastructural analyses. BioMetals.

[18] Kanu A.S., Ashraf U., Bangura A., Yang D.M., Ngaujah A.S., Tang X. (2017). Cadmium (Cd) Stress in Rice; Phyto-Availability, Toxic Effects, and Mitigation Measures-A Critical Review. IOSR-JESTFT.

[19] Liu J., Li K., Xu J., Liang J., Lu X., Yang J., Zhu Q. (2003). Interaction of Cd and five mineral nutrients for uptake and accumulation in different rice cultivars and genotypes. Field Crop. Res..

[20] Liu J.G., Liang J.S., Li K.Q., Zhang Z.J., Yu B.Y., Lu X.L., Yang J.C., Zhu Q.S. (2003). Correlations between cadmium and mineral nutrients in absorption and accumulation in various genotypes of rice under cadmium stress. Chemosphere.

[21] Rascio N., Dalla Vecchia F., La Rocca N., Barbato R., Pagliano C., Raviolo M., Gonnelli C., Gabbrielli R. (2008). Metal accumulation and damage in rice (*cv*. Vialone nano) seedlings exposed to cadmium. Environ. Exp. Bot..

[22] Yu H., Wang J., Fang W., Yuan J., Yang Z. (2006). Cadmiumaccumulation in different rice cultivars and screening for pollution safe cultivars of rice. Sci. Total Environ..

[23] Zhang X., Gao H., Zhang Z., Wan X. (2013). Influences of different ion channel inhibitors on the absorption of fluoride in tea plants (Camellia sinesis L.). Plant Growth Regul..

[24] Arao T., Ae N. (2003). Genotypic variations in cadmium levels of rice grain. Soil Sci. Plant Nutr..

[25] He J., Zhu C., Ren Y., Yan Y., Jiang D. (2006). Genotypic variation in grain cadmium concentration of lowland rice. J. Plant Nutr. Soil Sci..

[26] Liu H.J., Zhang J.L., Christie P., Zhang F.S. (2007). Influence of external zinc and phosphorus supply on Cd uptake by rice (*Oryza sativa* L.) seedlings with root surface iron plaque. Plant Soil..

[27] Rodda M.S., Li G., Reid R.J. (2011). The timing of grain Cd accumulation in rice plants: The relative importance of remobilisation within the plant and root Cd uptake post flowering. Plant Soil..

[28] Zhou H., Zhou X., Zeng M., Liao B.H., Liu L., Yang W.T., We Y.M., Qiu Q.Y., Wang Y.J. (2014). Effects of combined amendments on heavy metal accumulation in rice (*Oryza sativa* L.) planted on contaminated paddy soil. Ecotoxicol. Environ. Saf..

[29] Mostofa M.G., Rahman A., Ansary M.M.U., Watanabe A., Fujita M., Tran L.S.P. (2015). Hydrogen sulfide modulates cadmium-induced physiological and biochemical responses to alleviate cadmium toxicity in rice. Sci. Rep..

[30] Rehman M.Z., Rizwan M., Ghafoor A., Naeem A., Ali S., Sabir M., Qayyum M.F. (2015). Effect of inorganic amendments for in situ stabilization of cadmium in contaminated soils and its phyto-availability to wheat and rice under rotation. Environ. Sci. Pollut. Res..

[31] Shah K., Nahakpam S. (2012). Heat exposure alters the expression of SOD, POD, APX and CAT isozymes and mitigates low cadmium toxicity in seedlings of sensitive and tolerant rice cultivars. Plant Physiol. Biochem..

[32] Zhang C., Yin X., Gao K., Ge Y., Cheng W. (2013). Non-protein thiols and glutathione S-transferase alleviate Cd stress and reduce root-to-shoot translocation of Cd in rice. J. Plant Nutr Soil Sci..

[33] Choppala G., Saifullah M.F., Bolan N., Bibi S., Iqbal M., Rengel Z., Kunhikrishnan A., Ashwath N., Ok Y.S. (2014). Cellular mechanisms in higher plants governing tolerance to cadmium toxicity. Crit. Rev. Plant Sci..

[34] Yu C., Sun C., Shen C., Wang S., Liu F., Liu Y., Chen Y., Li C., Qian Q., Aryal B. (2015). The auxin transporter, OsAUX1, is involved in primary root and root hair elongation and in Cd stress responses in rice (*Oryza sativa* L.). Plant J..

[35] Zhang X., Wu H., Chen L., Liu L., Wan X. (2018). Maintenance of mesophyll potassium and regulation of plasma membrane H^+^-ATPase are associated with physiological responses of tea plants to drought and subsequent rehydration. Crop. J..

[36] Parrotta L., Guerriero G., Sergeant K., Cai G., Hausman J.F. (2015). Target or barrier? The cell wall of early- and later-diverging plants vs cadmium toxicity: Differences in the response mechanisms. Front. Plant Sci..

[37] Loix C., Huybrechts M., Vangronsveld J., Gielen M., Keunen E., Cuypers A. (2017). Reciprocal Interactions between Cadmium-Induced Cell Wall Responses and Oxidative Stress in Plants. Front. Plant Sci..

[38] Hall J.L. (2002). Cellular mechanisms for heavy metal detoxification and tolerance. J. Exp. Bot..

[39] Qiu Q., Wang Y.T., Yang Z.Y., Yuan J.G. (2011). Effects of phosphorus supplied in soil on subcellular distribution and chemical forms of cadmium in two Chinese flowering cabbage (Brassica parachinensis L.) cultivars differing in cadmium accumulation. Food Chem. Toxicol..

[40] Wang X., Liu Y.O., Zeng G.M., Chai L.Y., Song X.C., Min Z.Y., Xiao X. (2008). Subcellular distribution and chemical forms of cadmium in *Bechmeria nivea* (L.). Gaud. Environ. Exp. Bot..

[41] Zhang J., Sun W., Li Z., Liang Y., Song A. (2009). Cadmium fate and tolerance in rice cultivars. Agron. Sustain. Dev..

[42] Fu X.P., Dou C.M., Chen Y.X., Chen X.C., Shi J.Y., Yu M.G., Xu J. (2011). Subcellular distribution and chemical forms of cadmium in *Phytolacca americana* L.. J. Hazard. Mater..

[43] Kim D.Y., Bovet L., Maeshima M., Martinoia E., Lee Y.S. (2007). The ABC transporter AtPDR8 is a cadmium extrusion pump conferring heavy metal resistance. Plant J..

[44] Park J., Song W.Y., Ko D., Eom Y., Hansen T.H., Schiller M., Lee T.G., Martinoia E., Lee Y.S. (2012). The phytochelatin transporters AtABCC1 and AtABCC2 mediate tolerance to cadmium and mercury. Plant J..

[45] Oda K., Otani M., Uraguchi S., Akihiro T., Fujiwara T. (2011). Rice *ABCG43* is Cd inducible and confers Cd tolerance on yeast. Biosci. Biotech. Biochem..

[46] Sasaki A., Yamaji N., Ma J.F. (2014). Overexpression of *OsHMA3* enhances Cd tolerance and expression of Zn transporter genes in rice. J. Exp. Bot..

[47] Ueno D., Koyama E., Yamaji N., Ma J.F. (2011). Physiological, genetic, molecular characterization of a high-Cd-accumulating rice cultivar, Jarjan. J. Exp. Bot..

[48] Dong J., Mao W.H., Zhang G.P., Wu F.B., Cai Y. (2007). Root excretion and plant tolerance to cadmium toxicity—a review. Plant Soil Environ..

[49] Haydon M.J., Cobbett C.S. (2007). Transporters of ligands for essential metal ions in plants. New Phytol..

[50] Jabeen R., Ahmad A., Iqbal M. (2009). Phytoremediation of heavy metals: Physiological and molecular mechanisms. Bot. Rev..

[51] Verbruggen N., Hermans C., Schat H. (2009). Molecular mechanisms of metal hyperaccumulation in plants. New Phytol..

[52] Nocito F.F., Lancilli C., Dendena B., Lucchini G., Sacchi G.A. (2011). Cadmium retention in rice roots is influenced by cadmium availability, chelation and translocation. Plant Cell Environ..

[53] Hassan M.J., Shao G., Zhang G. (2005). Influence of cadmium toxicity on growth and antioxidant enzyme activity in rice cultivars with different grain cadmium accumulation. J. Plant Nutr..

[54] Hsu Y.T., Kao C.H. (2007). Cadmium-induced oxidative damage in rice leaves is reduced by polyamines. Plant Soil..

[55] Lin R., Wang X., Luo Y., Du W., Guo H., Yin D. (2007). Effects of soil cadmium on growth, oxidative stress and antioxidant system in wheat seedlings *Triticum aestivum* L.. Chemosphere.

[56] Roychoudhury A., Basu S., Sengupta D.N. (2012). Antioxidants and stressrelated metabolites in the seedlings of two indica rice varieties exposed to cadmium chloride toxicity. Acta Physiol. Plant.

[57] Shen G.M., Zhu C., Du Q.Z., Shangguan L.N. (2012). Ascorbate-glutathione cycle alteration in cadmium sensitive rice mutant *cadB1*. Rice Sci..

[58] Srivastava R.K., Pandey P., Rajpoot R., Rani A., Dubey R.S. (2014). Cadmium and lead interactive effects on oxidative stress and antioxidative responses in rice seedlings. Protoplasma.

[59] Asgher M., Khan M.I., Anjum N.A., Khan N.A. (2015). Minimising toxicity of cadmium in plants--role of plant growth regulators. Protoplasma.

[60] Aina R., Labra M., Fumagalli P., Vannini C., Marsoni M., Cucchi U., Bracale M., Sgorbati S., Citterio S. (2007). Thiol-peptide level and proteomic changes in response to cadmium toxicity in *Oryza sativa* L. roots. Environ. Exp. Bot..

[61] Chao Y.Y., Chen C.Y., Huang W.D., Kao C.H. (2010). Salicylic acidmediated hydrogen peroxide accumulation and protection against Cd toxicity in rice leaves. Plant Soil..

[62] Zhang C.H., Ying G.E. (2008). Response of glutathione and glutathione Stransferase in rice seedlings exposed to cadmium stress. Rice Sci..

[63] Lee K., Bae D.W., Kim S.H., Han H.J., Liu X., Park H.C., Lim C.O., Lee X.Y., Chung W.S. (2010). Comparative proteomic analysis of the shortterm responses of rice roots and leaves to cadmium. J. Plant Physiol..

[64] Khavari-Nejad R.A., Najafi F., Rezaei M. (2014). The influence of cadmium toxicity on some physiological parameters as affected by iron in rice (Oryza Sativa L.) plant. J. Plant Nutr..

[65] Wang M.Y., Chen A.K., Wong M.H., Qiu R.L., Cheng H., Ye Z.H. (2011). Cadmium accumulation in and tolerance of rice *Oryza sativa* L. varieties with different rates of radial oxygen loss. Environ. Pollut..

[66] Agrawal G.K., Rakwal R., Yonekura M., Kubo A., Saji H. (2002). Rapid induction of defense/stress-related proteins in leaves of rice (*Oryza sativa*) seedlings exposed to ozone is preceded by newly phosphorylated proteins and changes in a 66-kDA ERK-typeMAPK. J. Plant Physiol..

[67] Agrawal G.K., Rakwal R., Iwahashi H. (2002). Isolation of novel rice (*Oryza sativa* L.) multiple stress responsive MAP kinase gene, *OsMSRMK2*, whose mRNA accumulates rapidly in response to environmental cues. Biochem. Biophys. Res. Commun..

[68] Yeh C.M., Hsiao L.J.H., Hsiao H.J. (2004). Cadmium activates a mitogenactivated protein kinase gene and MBP kinases in rice. Plant Cell Physiol..

[69] Zhao F.Y., Hu F., Zhang S.Y., Wang K., Zhang C.R., Liu T. (2013). MAPKs regulate root growth by influencing auxin signaling and cell cyclerelated gene expression in cadmium-stressed rice. Environ. Sci. Pollut. Res..

[70] Wang X., Yao H., Wong M.H., Ye Z. (2013). Dynamic changes in radial oxygen loss and iron plaque formation and their effects on Cd and As accumulation in rice (*Oryza sativa* L.). Environ. Geochem. Health.

[71] Cheng H., Wang M., Wong M.H., Ye Z. (2014). Does radial oxygen loss and iron plaque formation on roots alter Cd and Pb uptake and distribution in rice plant tissues?. Plant Soil..

[72] Shao J.F., Che J., Yamaji N., Shen R.F., Ma J.F. (2017). Silicon reduces cadmium accumulation by suppressing expression of transporter genes involved in cadmium uptake and translocation in rice. J. Exp. Bot..

[73] Chen Z., Tang Y.T., Yao A.J., Cao J., Wu Z.H., Peng Z.R., Wang S.Z., Xiao S., Baker A.J.M., Qiu R.L. (2017). Mitigation of Cd accumulation in paddy rice (*Oryza sativa* L.) by Fe fertilization. Environ. Pollut..

[74] Huang G., Ding C., Zhou Z., Zhang T., Wang X. (2019). A tillering application of zinc fertilizer based on basal stabilization reduces Cd accumulation in rice (*Oryza sativa* L.). Ecotoxicol. Environ. Saf..

[75] Chen D., Chen D., Xue R., Long J., Lin X., Lin Y., Jia L., Zeng R., Song Y. (2019). Effects of boron, silicon and their interactions on cadmium accumulation and toxicity in rice plants. J. Hazard. Mater..

[76] Hermans C., Conn S.J., Chen J., Xiao Q., Verbruggen N. (2013). An update on magnesium homeostasis mechanisms in plants. Metallomics.

[77] Lee S., Kim Y.Y., Lee Y., An G. (2007). Rice P1B-type heavy-metal ATPase, OsHMA9, is a metal efflux protein. Plant Physiol..

[78] Shimo H., Ishimaru Y., An G., Yamakawa T., Nakanishi H., Nishizawa N.K. (2011). *Low cadmium* (*LCD*), a novel gene related to cadmium tolerance and accumulation in rice. J. Exp Bot..

[79] Kuramata M., Masuya S., Takahashi Y., Kitagawa E., Inoue C., Ishikawa S., Youssefian S., Kusano T. (2009). Novel cysteine-rich peptides from Digitaria ciliaris and Oryza sativa enhance tolerance to cadmium by limiting its cellular accumulation. Plant Cell Physiol..

[80] Yang J., Gao M.X., Hu H., Ding X.M., Lin H.W., Wang L., Xu J.M., Mao C.Z., Zhao F.J., Wu Z.C. (2016). OsCLT1, a CRT-like transporter 1, is required for glutathione homeostasis and arsenic tolerance in rice. New Phytol..

[81] Moons A. (2003). *Ospdr9*, which encodes a PDR-type ABC transporter, is induced by heavy metals, hypoxic stress and redox perturbations in rice roots. FEBS Lett..

[82] Agrawal G.K., Agrawal S.K., Shibato J., Iwahashi H., Rakwal R. (2003). Novel rice MAP kinases *OsMSRMK3* and *OsWJUMK1* involved in encountering diverse environmental stresses and developmental regulation. Biochem. Biophys. Res. Commun..

[83] Shim D., Jae-Ung H., Lee J., Lee S., Choi Y., An G., Martinoia E., Lee Y. (2009). Orthologs of the class A4 heat shock transcription factor HsfA4a confer cadmium tolerance in wheat and rice. Plant Cell..

[84] Ramesh S.A., Shin R., Eide D.J., Schachtman D.P. (2003). Differential metal selectivity and gene expression of two zinc transporters from rice. Plant Physiol..

[85] Chou T.S., Chao Y.Y., Huang W.D., Hong C.Y., Kao C.H. (2011). Effect of magnesium deficiency on antioxidant status and cadmium toxicity in rice seedlings. J. Plant Physiol..

[86] Yu L.H., Umeda M., Liu J.Y., Zhao N.M., Uchimiya H. (1998). A novel MT gene of rice plants is strongly expressed in the node portion of the stem. Gene.

[87] Cheng L., Wang F., Shou H., Huang F., Zheng L., He F., Li J., Zhao F.J., Ueno D., Ma J.F. (2007). Mutation in nicotianamine aminotransferase stimulated the Fe(II) acquisition system and led to iron accumulation in rice. Plant Physiol..

[88] Luo J.S., Huang J., Zeng D.L., Peng J.S., Zhang G.B., Ma H.L., Guan Y., Yi H.Y., Fu Y.L., Han B. (2018). A defensin-like protein drives cadmium efflux and allocation in rice. Nat. Commun..

[89] Masuda H., Ishimaru Y., Aung M.S., Kobayashi T., Kakei Y., Takahashi M., Higuchi K., Nakanishi H., Nishizawa N.K. (2012). Iron biofortification in rice by the introduction of multiple genes involved in iron nutrition. Sci. Rep..

[90] Yan H., Xu W., Xie J., Gao Y., Wu L., Sun L., Feng L., Chen X., Zhang T., Dai C. (2019). Variation of a major facilitator superfamily gene contributes to differential cadmium accumulation between rice subspecies. Nat. Commun..

[91] Zhao J., Yang W., Zhang S., Yang T., Liu Q., Dong J., Fu H., Mao X., Liu B. (2018). Genome-wide association study and candidate gene analysis of rice cadmium accumulation in grain in a diverse rice collection. Rice.

[92] Ansarypour Z., Shahpiri A. (2017). Heterologous expression of a rice metallothionein isoform (*OsMTI-1b*) in *Saccharomyces cerevisiae* enhances cadmium, hydrogen peroxide and ethanol tolerance. Braz. J. Microbiol..

[93] Ishimaru Y., Kakei Y., Shimo H., Bashir K., Sato Y., Sato Y., Uozumi N., Nakanishi H., Nishizawa N.K. (2011). A rice phenolic efflux transporter is essential for solubilizing precipitated apoplasmic iron in the plant stele. J. Biol. Chem..

[94] Hao X., Zeng M., Wang J., Zeng Z., Dai J., Xie Z., Yang Y., Tian L., Chen L., Li D. (2018). A Node-Expressed Transporter OsCCX2 Is Involved in Grain Cadmium Accumulation of Rice. Front. Plant Sci..

[95] Nakanishi H., Ogawa I., Ishimaru Y., Mori S., Nishizawa N.K. (2006). Iron deficiency enhances cadmium uptake and translocation mediated by the Fe^2+^ transporters OsIRT1 and OsIRT2 in rice. Soil Sci. Plant Nutr..

[96] Lee S., An G. (2009). Over-expression of *OsIRT1* leads to increased iron and zinc accumulations in rice. Plant Cell Environ..

[97] Yuan L., Yang S., Liu B., Zhang M., Wu K. (2012). Molecular characterization of a rice metal tolerance protein, OsMTP1. Plant Cell Rep..

[98] Kavitha P.G., Kuruvilla S., Mathew M.K. (2015). Functional characterization of a transition metal ion transporter, OsZIP6 from rice (*Oryza sativa* L.). Plant Physiol. Biochem..

[99] Tan L., Zhu Y., Fan T., Peng C., Wang J., Sun L., Chen C. (2019). *OsZIP7* functions in xylem loading in roots and inter-vascular transfer in nodes to deliver Zn/Cd to grain in rice. Biochem. Biophys. Res. Commun..

[100] Das N., Bhattacharya S., Bhattacharyya S., Maiti M. (2017). Identification of alternatively spliced transcripts of rice phytochelatin synthase 2 gene *OsPCS2* involved in mitigation of cadmium and arsenic stresses. Plant Mol. Biol..

[101] Uraguchi S., Kamiya T., Sakamoto T., Kasai K., Sato Y., Nagamura Y., Yoshida A., Kyozuka J., Ishikawa S., Fujiwara T. (2011). Low-affinity cation transporter (*OsLCT1*) regulates cadmium transport into rice grains. Proc. Natl. Acad Sci..

[102] Uraguchi S., Kamiya T., Clemens S., Fujiwara T. (2014). Characterization of OsLCT1, a cadmium transporter from indica rice *Oryza sativa*. Physiol. Plant.

[103] Takahashi R., Ishimaru Y., Shimo H., Ogo Y., Senoura T., Nishizawa N.K., Nakanishi H. (2012). The OsHMA2 transporter is involved in root-to-shoot translocation of Zn and Cd in rice. Plant Cell Environ..

[104] Yamaji N., Xia J., Mitani-Ueno N., Yokosho K., Ma J.F. (2013). Preferential delivery of zinc to developing tissues in rice is mediated by P-type heavy metal ATPase OsHMA2. Plant Physiol..

[105] Ueno D., Yamaji N., Kono I., Huang C.F.T., Yano M., Ma J.F. (2010). Gene limiting cadmium accumulation in rice. Proc. Natl. Acad Sci. USA.

[106] Ishimaru Y., Takahashi R., Bashir K., Shimo H., Senoura T., Sugimoto K., Ono K., Yano M., Ishikawa S., Arao T. (2012). Characterizing the role of rice NRAMP5 in manganese, iron and cadmium transport. Sci. Rep..

[107] Sasaki A., Yamaji N., Yokosho K., Ma J.F. (2012). Nramp5 is a major transporter responsible for manganese and cadmium uptake in rice. Plant Cell..

[108] Yang M., Zhang Y.Y., Zhang L., Hu J., Zhang X., Lu K., Dong H., Wang D., Zhao F.J., Huang C.F. (2014). OsNRAMP5 contributes to manganese translocation and distribution in rice shoots. J. Exp. Bot..

[109] Takahashi R., Ishimaru Y., Shimo H., Bashir K., Senoura T., Sugimoto K., Ono K., Suzui N., Kawachi N., Ishii S. (2014). From laboratory to field: *OsNRAMP5*-knockdown rice is a promising candidate for Cd phytoremediation in paddy fields. PLoS ONE.

[110] Tang L., Mao B., Li Y., Lv Q., Zhang L., Chen C., He H., Wang W., Zeng X., Shao Y. (2017). Knockout of *OsNramp5* using the CRISPR/Cas9 system produces low Cd-accumulating indica rice without compromising yield. Sci. Rep..

[111] Takahashi R., Ishimaru Y., Nakanishi H., Nishizawa N.K. (2011). Role of the iron transporter OsNRAMP1 in cadmium uptake and accumulation in rice. Plant Signal. Behav..

[112] Takahashi R., Ishimaru Y., Senoura T., Shimo H., Ishikawa S., Arao T., Nakanishi H., Nishizawa N.K. (2011). The OsNRAMP1 iron transporter is involved in Cd accumulation in rice. J. Exp. Bot..

[113] Tiwari M., Sharma D., Dwivedi S., Singh M., Tripathi R.D., Trivedi P.K. (2014). Expression in Arabidopsis and cellular localization reveal involvement of rice NRAMP, OsNRAMP1, in arsenic transport and tolerance. Plant Cell Environ..

[114] Lim S.D., Hwang J.G., Han A.R., Park Y.C., Lee C., Ok Y.S., Jang C.S. (2014). Positive regulation of rice RING E3 ligase OsHIR1 in arsenic and cadmium uptakes. Plant Mol. Biol..

[115] 125Mukhopadhyay A., Vij S., Tyagi A.K. (2004). Overexpression of a zinc-finger protein gene from rice confers tolerance to cold, dehydration, and salt stress in transgenic tobacco. Proc. Natl. Acad. Sci. USA.

[116] Wang F.J., Wang M., Liu Z.P., Shi Y., Han T.Q., Ye Y.Y., Gong N., Sun J.W., Zhu C. (2015). Different responses of low grain-Cd-accumulating and high grain-Cd-accumulating rice cultivars to Cd stress. Plant Mol. Biol..

[117] Jin S., Cheng Y., Guan Q., Liu D., Takano T., Liu S. (2006). A metallothionein-like protein of rice (rgMT) functions in *E. coli* and its gene expression is induced by abiotic stresses. Biotechnol. Lett..

[118] Harada E., Choi Y.E., Tsuchisaka A., Obata H., Sano H. (2001). Transgenic tobacco plants expressing a rice cysteine synthase gene are tolerant to toxic levels of cadmium. J. Plant Physiol..

[119] Lu L.L., Tian S.K., Yang X.E., Li T.Q., He Z.L. (2009). Cadmium uptake and xylem loading are active processes in the hyperaccumulator Sedum alfredii. J. Plant Physiol..

[120] Ishimaru Y., Suzuki M., Tsukamoto T., Suzuki K., Nakazono M., Kobayashi T., Wada Y., Watanabe S., Matsuhashi S., Takahashi M. (2006). Rice plants take up iron as an Fe^3+^-phytosiderophore and as Fe^2+^. Plant J..

[121] Liu C., Chen G., Li Y., Peng Y., Zhang A., Hong K., Jiang H., Ruan B., Zhang B., Yang S. (2017). Characterization of a major QTL for manganese accumulation in rice grain. Sci. Rep..

[122] Uraguchi S., Fujiwara T. (2012). Cadmium transport and tolerance in rice: Perspectives for reducing grain cadmium accumulation. Rice.

[123] Satoh-Nagasawa N., Mori M., Nakazawa N., Kawamoto T., Nagato Y., Sakurai K., Takahashi H., Watanabe A., Akagi H. (2012). Mutations in rice (*Oryza sativa*) heavy metal ATPase 2 (*OsHMA2*) restrict the translocation of zinc and cadmium. Plant Cell Physiol..

[124] Satoh-Nagasawa N., Mori M., Sakurai K., Takahashi H., Watanabe A., Akagi H. (2013). Functional relationship heavy metal P-type ATPases (*OsHMA2* and *OsHMA3*) of rice (*Oryza sativa*) using RNAi. Plant Biotechnol..

[125] Miyadate H., Adachi S., Hiraizumi A., Tezuka K., Nakazawa N., Kawamoto T., Katou K., Kodama I., Sakurai K., Takahashi H. (2011). OsHMA3, a P1B-type of ATPase affects root-to-shoot cadmium translocation in rice by mediating efflux into vacuoles. New Phytol..

[126] Tanaka K., Fujimaki S., Fujiwara T., Yoneyama T., Hayashi H. (2007). Quantitative estimation of the contribution of the phloem in cadmium transport to grains in rice plants (Oryza sativa L.). Soil Sci. Plant Nutr..

[127] Wu Z.C., Zhao X.H., Sun X.C., Tan Q.L., Tang Y.F., Nie Z.J., Hu C.X. (2015). Xylem transport and gene expression play decisive roles in cadmium accumulation in shoots of two oilseed rape cultivars (Brassica napus). Chemosphere.

[128] Fujimaki S., Suzui N., Ishioka N.S., Kawachi N., Ito S., Chino M., Nakamura S. (2010). Tracing cadmium from culture to spikelet: Noninvasive imaging and quantitative characterization of absorption, transport, and accumulation of cadmium in an intact rice plant. Plant Physiol..

[129] Yan J., Wang P., Wang P., Yang M., Lian X., Tang Z., Huang C.F., Salt D.E., Zhao F.J. (2016). A loss-of-function allele of *OsHMA3* associated with high cadmium accumulation in shoots and grain of Japonica rice cultivars. Plant Cell Environ..

[130] Ishikawa S., Abe T., Kuramata M., Yamaguchi M., Ando T., Yamamoto T., Yano M. (2010). A major quantitative trait locus for increasing cadmium-specific concentration in rice grain is located on the short arm of chromosome 7. J. Exp. Bot..

[131] Kashiwagi T., Shindoh K., Hirotsu N., Ishimaru K. (2009). Evidence for separate translocation pathways in determining cadmium accumulation in grain and aerial plant parts in rice. BMC Plant Biol..

[132] Sato H., Shirasawa S., Maeda H., Nakagomi K., Kaji R., Ohta H., Yamaguchi M., Takeshi N. (2011). Analysis of QTL for lowering cadmium concentration in rice grains from ‘LAC- 23′. Breed. Sci..

[133] Yan Y.F., Lestari P., Lee K.J., Kim M.Y., Lee S.H., Lee B.W. (2013). Identification of quantitative trait loci for cadmi um accumulation and distribution in rice (Oryza sativa). Genome.

[134] Abe T., Nonoue Y., Ono N., Omoteno M., Kuramata M., Fukuoka S., Yamamoto T., Yano M., Ishikawa S. (2013). Detection of QTLs to reduce cadmium content in rice grains using LAC23/Koshihikari chromosome segment substitution lines. Breed. Sci..

[135] Liu W., Pan X., Li Y., Duan Y., Min J., Liu S., Liu L., Sheng X., Li X. (2019). Identification of QTLs and Validation of *qCd-2* Associated with Grain Cadmium Concentrations in Rice. Rice Sci..

[136] Liu X., Chen S., Chen M., Zheng G., Peng Y., Shi X., Qin P., Xu X., Teng S. (2019). Association Study Reveals Genetic Loci Responsible for Arsenic, Cadmium and Lead Accumulation in Rice Grain in Contaminated Farmlands. Front. Plant Sci..

[137] Ueno D., Koyama E., Kono I., Ando T., Yano M., Ma J.F. (2009). Identification of a novel major quantitative trait locus controlling distribution of Cd between roots and shoots in rice. Plant Cell Physiol..

[138] Ueno D., Kono I., Yokosho K., Ando T., Yano M., Ma J.F. (2009). A major quantitative trait locus controlling cadmium translocation in rice (*Oryza sativa*). New Phytol..

[139] Xue D., Chen M., Zhang G. (2009). Mapping of QTLs associated with cadmium tolerance and accumulation during seedling stage in rice (*Oryza sativa* L.). Euphytica.

[140] Norton G.J., Deacon C.M., Xiong L.Z., Huang S.Y., Meharg A.A., Price A.H. (2010). Genetic mapping of the rice ionome in leaves and grain: Identification of QTLs for 17 elements including arsenic, cadmium, iron and selenium. Plant Soil.

[141] Ishikawa S., Ae N., Yano M. (2005). Chromosomal regions with quantitative trait loci controlling cadmium concentration in brown rice (*Oryza sativa*). New Phytol..

[142] Abe T., Taguchi-Shiobara F., Kojima Y., Ebitani T., Kuramata M., Yamamoto T., Yano M., Ishikawa S. (2011). Detection of a QTL for accumulating Cd in rice that enables efficient Cd phytoextraction from soil. Breed. Sci..

[143] Zhang X.Q., Zhang G.P., Guo L.B., Wang H.Z., Zeng D.L., Dong G.J., Qian Q., Xue D.W. (2011). Identification of quantitative trait loci for Cd and Zn concentrations of brown rice grown in Cd-polluted soils. Euphytica.

[144] Huang Y., Sun C., Min J., Chen Y., Tong C., Bao J. (2015). Association Mapping of Quantitative Trait Loci for Mineral Element Contents in Whole Grain Rice (*Oryza sativa* L.). J. Agric. Food Chem..

[145] Guo G., Zhou Q., Ma L.Q. (2006). Availability and assessment of fixing additives for the in-situ remediation of heavy metal contaminated soils: A review. Environ. Monit. Assess..

[146] Madejón E., de Mora A.P., Felipe E., Burgos P., Cabrera F. (2006). Soil amendments reduce trace element solubility in a contaminated soil and allow regrowth of natural vegetation. Environ. Pollut..

[147] Yan Y., Zhou Y.Q., Liang C.H. (2015). Evaluation of phosphate fertilizers for the immobilization of Cd in contaminated soils. PLoS ONE.

[148] Hu P.J., Ouyang Y.N., Wu L.H., Shen L.B., Luo Y.M., Christie P. (2015). Effects of water management on arsenic and cadmium speciation and accumulation in an upland rice cultivar. J. Environ. Sci..

[149] Honma T., Ohba H., Kaneko-Kadokura A., Makino T., Nakamura K., Katou H. (2016). Optimal soil Eh, pH, and water management for simultaneously minimizing arsenic and cadmium concentrations in rice grains. Environ. Sci. Technol..

[150] Yu L.L., Zhu J.Y., Huang Q.Q., Su D.C., Jiang R.F., Li H.F. (2014). Application of a rotation system to oilseed rape and rice fields in Cd-contaminated agricultural land to ensure food safety. Ecotox. Environ. Safe..

[151] Ishikawa S., Ishimaru Y., Igura M., Kuramata M., Abe T., Senoura T., Hase Y., Arao T., Nishizawa N.K., Nakanishi H. (2012). Ion-beam irradiation, gene identification, and marker-assisted breeding in the development of low-cadmium rice. Proc. Natl. Acad. Sci. USA.

[152] Lu C., Zhang L., Tang Z., Huang X.Y., Ma J.F., Zhao F.J. (2019). Producing cadmium-free Indica rice by overexpressing *OsHMA3*. Environ. Int..

